# Latent infection of human bocavirus accompanied by flare of chronic cough, fatigue and episodes of viral replication in an immunocompetent adult patient, Cologne, Germany

**DOI:** 10.1099/jmmcr.0.005052

**Published:** 2016-08-30

**Authors:** Wolfram Windisch, Monika Pieper, Inga Ziemele, Jürgen Rockstroh, Michael Brockmann, Oliver Schildgen, Verena Schildgen

**Affiliations:** ^1^​ Department of Pneumology, Kliniken der Stadt Köln gGmbH, University of Witten/Herdecke, Cologne, Germany; ^2^​ Institut für Pathologie, Klinikum der Privaten Universität Witten/Herdecke mit Sitz in Köln, Cologne, Germany; ^3^​ Virology, University of Helsinki, Helsinki, Finland; ^4^​ Riga Stradiņš University, Riga, Latvia; ^5^​ Universitätsklinikum Bonn, Immunologische Ambulanz, Bonn, Germany

**Keywords:** human bocavirus, chronic persistence, chronic cough

## Abstract

**Introduction:**

The human bocavirus (HBoV) is a parvovirus and is associated with mild to life-threatening acute or persisting respiratory infections, frequently accompanied by further pathogens. So far, there is limited knowledge on the mechanisms of persistence, and no reports on chronic infections or latency have been published so far.

**Case presentation:**

An immunocompetent male patient suffers from a chronic HBoV1 infection, i.e. viral DNA was detected in both serum and bronchoalveolar lavage (BAL) for >5 months without co-infections and with respiratory symptoms resolved spontaneously while receiving symptomatic treatment with montelukast and corticosteroids. Following the symptomatic medication of a chronic infection with HBoV1 viraemia indicating active viral replication lasting over 5 months, the patient cleared the viraemia and no further viral DNA was detectable in the BAL. However, by fluorescence *in situ* hybridization analyses of mucosal biopsies, it was shown that the virus genome still persisted in the absence of viral shedding but in a more compact manner possibly representing a supercoiled episomal form of this otherwise linear single-stranded DNA genome. This indicated the entry into a latency phase. Moreover, the cytokine profile and the IP-10/TARC ratio, a marker for fibrotization, seem to have been altered by HBoV1 replication. Although specific IgG antibodies were detectable during the whole observation period, they showed an apparently insufficient neutralising activity.

**Conclusion:**

On the one hand, these findings suggest that the symptomatic medication may have led to clearance of the virus from blood and airways and, moreover, that the viral DNA persists in the tissue as an altered episomal form favoured by lacking neutralising antibodies. This appears to be important in order to reduce possible long-term effects such as lung fibrosis.

## Introduction

In the majority of clinical studies and single cases described and observed so far, the human bocavirus (HBoV) type 1 HBoV1 was associated with mild to severe acute or persisting respiratory infections and symptoms ranging from the common cold to life-threatening pneumonia, mainly in children but occasionally also in adults (Allander, 2008; Garcia-Garcia *et al.*, 2010).

Because no animal model is available so far due to the narrow host range of the virus, clinical cases, besides *in vitro* studies in cell cultures, remain the major source of information about the pathogenesis of this virus. One feature of HBoV appears to be its ability to establish a latent stage or even chronic infection (Byington *et al.*, 2015; Kaur *et al.*, 2014; Schildgen *et al.*, 2013; Windisch *et al.*, 2013; Deng *et al.*, 2014; Manning *et al.*, 2007; Schenk *et al.*, 2011). Therefore, the presented case is of major importance; as for the first time, we describe an HBoV1-associated chronic respiratory infection in detail, provide evidence for clinical improvement following symptomatic treatment, gain insight into the molecular mechanisms of viral persistence and immune evasion of the virus and provide evidence that the chronic infection with HBoV can include flares of active viral replication followed by symptom free latency.

## Case report

The patient was a 29-year-old never-smoker Caucasian male, who came to our out-patient clinic because of chronic cough of so far unknown origin resistant to previous antimicrobiotic treatment. When presenting first in May 2013, the patient had symptoms of a persisting pharyngitis; from this time point on, he suffered from a worsening of his cough, which became chronic and persisted until January 2015. In addition, the patient described chronic fatigue that prevents the patient from practising his profession as a percussionist.

Bronchoscopy confirmed an acute tracheobronchitis in February and June 2014. Thereby, macroscopically mucosal lesions in the hypopharynx were observed in June 2014, and a biopsy was taken in June 2014.

In February 2014, a mild alveolitis with increased lymphocyte subsets was confirmed in the BAL, and in both BALs from February and June 2014, HBoV1-DNA (>10^5^ copies per millilitre) was detected by quantitative PCR (qPCR) in both cases accompanied by a viraemia verified by detection of viral DNA in corresponding serum samples independently in Cologne and Helsinki. The copy numbers in the sera were tested positive by qPCR in Cologne with 1.5×10^3^ (February) copies per millilitre and 1.8×10^3^ copies per millilitre (June). Furthermore, a myocardiac biopsy was taken in May 2014, which displayed a recently resolved myocarditis.

Due to the long and ongoing clinical symptoms, the patient was tested for an either primary or secondary immunodeficiency syndrome but neither an HIV infection nor any other immunodeficiency was diagnosed, whereas a combined type IIb hyperlipoproteinemia according to Fredrickson and a leaky gut syndrome were diagnosed.

## Investigations

Laboratory testing of the patient’s BAL included routine screening for respiratory bacteria by conventional microbiological screening methods and by culturing. In addition, the BALs were analysed for facultative and obligate respiratory bacteria as well as viruses and fungi by the Respifinder Smart 22 and Meningofinder Custom Assays (i.e. the standard Meningofinder plus mumps and measles probes, Pathofinder). The Respifinder and Meningofinder assays were previously described as suitable and sensitive tools by our group and others (Kaur *et al.*, 2014; Hardt *et al.*, 2014; Wolffs *et al.*, 2009; Dabisch-Ruthe *et al.*, 2012; Raymaekers *et al.*, 2011). In detail, the patient’s BAL was tested for influenza viruses, parainfluenza viruses 1–4, RSV, HMPV, HBoV1, coronaviruses NL63, OC43, 229E and HKU-1, adenoviruses, *Mycoplasma pneumoniae*, human parechoviruses, rhinoviruses and enteroviruses*, Legionella pneumoniae, Chlamydia pneumoniae* and *Bordetella pertussis* by Respifinder; mumps, measles, herpes simplex 1, herpes simplex 2, varicella zoster virus, Epstein Barr virus, cytomegalovirus, enterovirus and parechovirus by Meningofinder; *Pneumocystis jirovecii* by PCR (Schildgen *et al.*, 2014); and *Aspergillus* by ELISA (BioRad). Human herpesviruses 1–8 were also tested by qPCR as previously described (Windisch *et al.*, 2013). In addition, qPCR for quantification of HBoV1 was performed as previously described using the Qiagen QuantiTect Sybr Green Kit (Qiagen) using primers HBoV-Ku1 and HBoV-Ku2 followed by melting curve analyses (Kaur *et al.*, 2014; Kupfer *et al.*, 2006). *Mycobacteria* were tested by the MYCO-Direct 1.7 assay (Chipron). Routine culturing, including culturing of anaerobic pathogens, was performed by a microbiology laboratory. The laboratory results for viruses were further confirmed by the University Hospital Bonn, where also an immunodeficiency was excluded and normal immune cell counts and antibody titres were confirmed.

So far, as there is limited information about the local inflammation processes during an HBoV infection *in vivo*, we decided to retrospectively test the two BAL fluids of which aliquots were archived at −80 °C for the presence of cytokines, and we compared them to a BAL sampled in January 2015, in which no HBoV1-DNA was detected anymore. Therefore, the Abcam Cytokine Human Membrane Antibody Array (80 Targets, ab133998) was used as previously described (Khalfaoui *et al.*, 2016). The method detects the cytokines ENA-78, GCSF, GM-CSF, GRO, GRO-alpha, I-309, IL-1alpha, IL-1beta, IL-2, IL-3, IL-4, IL-5, IL-6, IL-7, IL-8, IL-10, IL-12 p40/p70, IL-13, IL-15, IFN-gamma, MCP-1, MCP-2, MCP-3, MCSF, MDC, MIG, MIP-1beta, MIP-1delta, RANTES, SCF, SDF-1, TARC, TGF-beta1, TNF-alpha, TNF-beta, EGF, IGF-I, Angiogenin, Oncostatin M, Thrombopoietin, VEGF-A, PDGF-BB, Leptin, BDNF, BLC, Ckß8-1, Eotaxin, Eotaxin-2, Eotaxin-3, FGF-4, FGF-6, FGF-7, FGF-9, Flt-3 Ligand, Fractalkine, GCP-2, GDNF, HGF, IGFBP-1, IGFBP-2, IGFBP-3, IGFBP-4, IL-16, IP-10, LIF, LIGHT, MCP-4, MIF, MIP-3 alpha, NAP-2, nt-3, nt-4, Osteopontin, Osteoprotegerin, PARC, PLGF, TGF-beta2, TGF-beta3, TIMP-1 and TIMP-2, respectively.

Moreover, the hypopharyngeal biopsies were tested for intracellular HBoV1-DNA by fluorescence *in situ* hybridization (FISH) as previously described (Schildgen *et al.*, 2013). HBoV1-specific IgM and IgG antibodies were assayed in serum samples obtained in February and June 2014 by competitive enzyme immunoassay (EIA) (Kantola *et al.*, 2011). The ODs measured in this ELISA were 1.589 for HBoV-1 IgG and 0.049 for HBoV-1 IgM in February, followed by 1.625 HBoV-1 IgG and 0.051 for HBoV-1 IgM in June, both indicating a past immune reaction.

In both BAL samples from February and June 2014, all tested pathogens that could have contributed to the clinical symptoms were repeatedly excluded except HBoV1, whose DNA was repeatedly detected by the Respifinder. The corresponding sera were tested positive at the analytic borderline of both the qPCR and the Respifinder assay performed in Cologne and below the limit of detection in the qPCR performed in Helsinki. Furthermore, the patient had medium EIA absorbances (1.639 and 1.264) of HBoV1-specific IgG antibodies with high avidity at both serum-collection points but was negative for IgM, indicating past immunity.

So far, it was shown by Kapoor *et al.* and our group that the HBoV-DNA can occur not only as a single-stranded DNA molecule but also as a covalently closed circular DNA (Kapoor *et al.*, 2011; Lusebrink *et al.*, 2011). Thereby, the Right-End-Hairpin sequence represents the ‘tail’-sequence while the Left-End-Hairpin represents the ‘head’-sequence (Schildgen *et al.*, 2012).

**Fig. 1.**
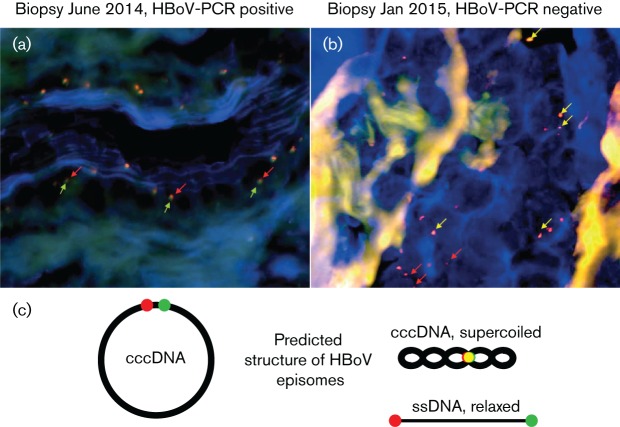
This figure shows the FISH analysis of biopsies of respiratory mucosa sampled in June 2014 (a) during the phase of active HBoV replication and in January 2015 (b) when blood and BAL were HBoV-DNA negative. Red signals show the HBoV genome’s tail, and green signals show the genome’s head. The arrows indicate the signals. It is obvious that, in June 2014, the signals were nearby each other, indicating an episomal form of the viral genome as described earlier by Kapoor *et al.* and our group (Kapoor *et al.*, 2011; Lusebrink *et al.*, 2011) but even more condensed in January 2015 when no viral shedding was observed anymore, thus maybe indicating a phase of latency. Panel (c) shows the putative DNA structures and the localization of the FISH signals in the cccDNA/episomal DNA, the condensed/supercoiled form and the relaxed single-stranded genome. The cccDNA thereby corresponds to panel A where active replication was observed, while the supercoiled form corresponds to panel B where no active replication was observed.

Besides the exclusive detection of HBoV-DNA in BAL and serum in February and June 2014, viral episomes were detected in the hypopharyngeal biopsy from June 2014 (Fig. 1a). These episomal structures were still present in the second biopsy from January 2015 (Fig. 1b), although the viraemia was cleared and the macroscopic inflammation signs were no longer present at that moment; thus, in January 2015, an objective improvement was clinically measurable, although the virus still persisted in the mucosal biopsies. Surprisingly, the HBoV FISH signals in the biopsy from January had a different shape compared to the first biopsy taken in June 2014. In June 2014, both the head (green) and the tail (red) signals were adjacent to each other but clearly divided, whereas in January 2015, the head and tail signals show an overlay and appear as yellow signals (Fig. 1b). Additionally, in some cases, single red signals occur. The most likely structural explanation for the FISH result is shown in Fig. 1c, which shows a scheme how the red and green FISH signals are localized on the viral genomic DNA in its putative forms (Fig. 1c).

**Fig. 2.**
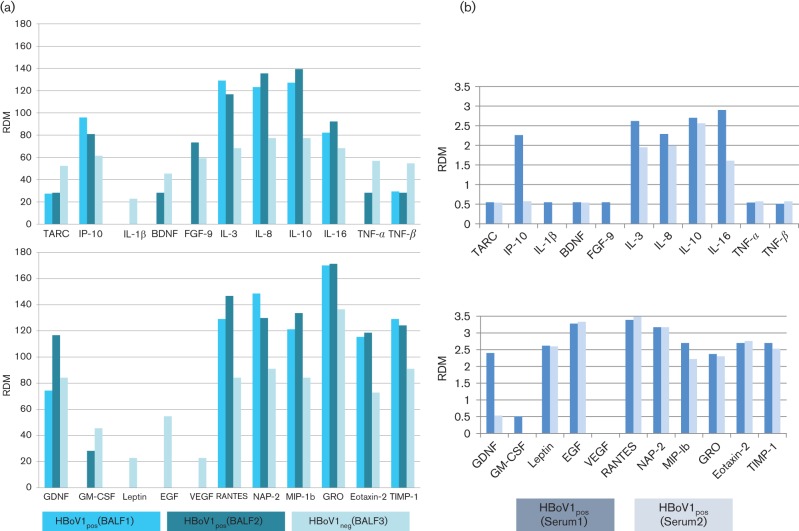
(a) Cytokine profile of the BAL sampled during and after the active viraemia. The first BAL was taken in February 2014, the second BAL was taken in June 2014 and the final BAL was taken in January 2015. The first two BALs were HBoV-positive by the Respifinder assay and qPCR, respectively, while the third BAL from January 2015 was tested negative for HBoV-DNA. The corresponding sera were also positive for HBoV-DNA in February 2014 and June 2014 but negative in January 2015. (b) Cytokine profiles of the sera corresponding to BAL 1 and BAL 2, respectively. In both cases, it was tested positive for HBoV by the Respifinder assay. No serum was available for the last BAL, which was negative for HBoV.

Simultaneously with the disappearance of HBoV1-DNA from BAL and serum, the majority of cytokines was downregulated in the BAL, such as RANTES, IL-3, IL-8, IL-10, NAP-2, TIMP-1 and IP-10 (Fig. 2), while expressions of RANTES, NAP-2, Eotaxin-2, TIMP-1, TNF-α and TNF-β remain at the same level in the HBoV-negative compared to the HBoV-positive serum. Moreover, the TARC/IP-10 increased during the HBoV infection in BAL (BAL_Feb14_ : 0.28; BAL_Jun14_ : 0.34; BAL_Jan15_ : 0.85) as well as in the serum (SER_Jun14_ : 0.23; SER_Jan15_ : 0.92), even when the viral DNA disappeared in BAL and serum. Altogether, the cytokine profiles observed in the stage of active replication followed by vanishing of the virus resemble the cytokine profile for chronic HBoV infections we previously described for a patient cohort and infected CuFi-8 cell cultures (Khalfaoui *et al.*, 2016).

## Diagnosis

The sole pathogen detected was the HBoV as described in the case presentation.

## Treatment

The patient was treated with montelukast and inhalative budesonide for 6 months (from July 2014 to January 2015) (Cai *et al.*, 2012) and subsequently controlled in January 2015. In this control check, the macroscopic mucosal lesions were not observed anymore, the patient displayed no objective signs of an airway infection or inflammation of respiratory epithelia, no pathogens were detected anymore in the BAL and the patient’s serum tested negative for HBoV1-DNA. This check-up was accompanied by sampling of a follow-up hypopharyngeal-bronchial biopsy also showing a recurrent finding.

## Outcome and follow-up

At the end of the observation period of our hospital, the patient appeared in increasing clinical condition before he changed to another ambulant care.

## Discussion

The present case is indeed important as it is a further confirmation of the hypothesis that HBoV is able to persist, also in otherwise healthy patients; beyond this, the present case report allows the conclusion that the virus not only can enter a phase of latency but also can actively replicate, the latter phase being accompanied by clinical symptoms. Here, not only the virus was shed for an unusual long time in the airways as already described by others (Martin *et al.*, 2010; Martin *et al.*, 2015; Byington *et al.*, 2015) but also the otherwise short-lived viraemia lasted here over 5 months. Additionally, FISH staining revealed most likely an episomal DNA structure in the respiratory mucosa, which could display a persisting form of the virus. The DNA occurred in different shapes that may be compatible with the replication stage. In June 2014, both the head signal and the tail signal were adjacent to each other but clearly divided, indicating a relaxed form of the circular DNA, maybe due to active viral replication, whereas in January 2015, the signals show an overlay and appear as yellow signals possibly indicating a supercoiled form that is not actively replicating. Some single red signals were also detected, which presumably represent deletion forms of the viral genome. The FISH results are consistent with the fact that, in January 2015, both the viraemia and the BAL were cleared, while the previous investigations were both tested positive for HBoV1-DNA.

Although the detected viraemia was weak, there is a high likelihood that HBoV contributed to the clinical symptoms as the virus was the single respiratory pathogen detected in the patient. One can assume that HBoV1 replication took place topical in the mucosal tissue where the viral DNA persisted, what is supported by the fact that HBoV also replicates in foci in cell culture (Dijkman *et al.*, 2009). This would also explain the low-level DNA amounts in asymptomatic blood donors, in whom viral DNA could circulate in low amounts as long as the virus persists and infected cells would be released to the blood stream (Bonvicini *et al.*, 2011).

Moreover, the patient displayed the persistent viraemia despite the presence of specific antibodies. Although the corticosteroids therapy could have transiently affected the immune system, it must be taken into account that, it may be possible that, HBoV1 does not induce neutralising antibodies during the infection or triggers a so far unknown immune-escape mechanism.

Besides, it is worth noting that the patient had a leaky gut syndrome as well as a history of a myocarditis from which he recovered because, in previous HBoV cases, we have detected HBoV-DNA post-mortem also in the myocardic tissue in patients that died from idiopathic lung fibrosis (Windisch *et al.*, 2013); thus, a causal link is possible and requires further attention. Although no clinical symptoms could be associated to HBoV detection, further reports of HBoV-DNA detection in the heart tissue exist (Kuethe *et al.*, 2009). In concert with the fact that myocardial infections are frequently observed after common colds in patients that were physically active during their common cold, HBoV could be accused being a further player in this clinical scenario. Consequently, as the leaky gut syndrome could not be deduced to other factors such as diet, inherited diseases or simultaneous infections, further studies should investigate on the role of HBoV in this syndrome because it could also be linked to the HBoV infection as HBoV is widely detected in feces (Albuquerque *et al.*, 2007; Lee *et al.*, 2007; Campe *et al.*, 2008; Cheng *et al.*, 2008; Yu *et al.*, 2008; Szomor *et al.*, 2009).

As previously discussed, the Th2 response in the lung is accompanied by increased expression levels of IL-4, IL-5, IL-10 and IL-13 and is followed by increased levels of, among others, TARC and RANTES (Berin *et al.*, 2001; Culley *et al.*, 2006). Also in the present case, an increased expression of RANTES and IL-10 besides IL-3, IL-8 and IL-16 was observed in the acute replication period. In contrast, the comparison of sera and BALs reveals a delayed tissue specific increase of Leptin, EGF, VEGF, TNF-α and TNF-β, which are directly or indirectly involved in fibrosing processes. Moreover, the HBoV-specific cytokine profile in the BAL is characterized by an imbalanced TARC/IP-10 ratio leading to the hypothesis that HBoV influences the Th1/Th2 response within the lung. Furthermore, an imbalanced TARC/IP-10 ratio seems to be associated with fibrotic lung diseases and it is worth to mention that the neutralization of TARC leads to a reduction of fibrosis in the animal (Keane, 2008; Belperio *et al.*, 2004). Despite the growing number of hints, more HBoV-positive follow-up cases have to be analysed to confirm HBoV-specific immune response modulation leading to chronic lung diseases.

However, the patient was successfully treated, and although not having lost the virus from the mucosal biopsies, he objectively recovered from the clinical symptoms and, as a matter of evidence-based speculation, the virus replaced active replication with a latency phase, as indicated by the conformational change observed in the FISH analysis.
